# Supplementary material for: Content-rich biological network constructed by mining PubMed abstracts
Source: BMC Bioinformatics. 2004 Oct 8;5:147. doi: 10.1186/1471-2105-5-147 (PMC528731; doi:10.1186/1471-2105-5-147)
Supplement: Additional File 2 — The original results of the above study (non-essential files are deleted to keep the file size under the limit set by BMC bioinformatics). [file 1471-2105-5-147-S2.bz2 › chilibotAdditionalFile2/dip05/685ID8980235E18004/html/IHFA_IHFB.html]

 


 **IHFA** and **IHFB** 
  
Found 14 abstracts in PubMed, retrieved 05.  
 

 What does Google say? 
 PDF only 
| .edu only 

---

**Interactive relationship** (e.g. stimulation, inhibition, etc)

**Non-interactive relationship** (e.g. studied together, co-existance, homology, etc.)

- In strain MG1655, mutants containing lesions in either the alpha  **ihfA**  or beta  **ihfB**  subunits of IHF show a marked decrease in both FimB 100 fold and FimE 1 00 fold promoted switching.  Ref: 9157242 Mol Microbiol, 1997
- Northern blotting and primer extension analysis defined the tsp for each gene and indicated a disparity in  **ihfA**  and  **ihfB**  message levels over time, with  **ihfB**  mRNA being more abundant throughout the entire growth cycle.  Ref: 9714829 Gene, 1998
- Also, the increase in expression was more pronounced when the IHF protein was mutated in the  **ihfB**  gene than in the  **ihfA**  gene.  Ref: 9735316 Plasmid, 1998
- In attempts to identify subunit specific phenotypes of ihf mutants we analyzed viability, thermoresistance and protein synthesis patterns in  **ihfA**  and  **ihfB**  mutants and their respective parental strains.  Ref: 9924977 Biochimie, 1998
- Furthermore, both the  **ihfA**  and  **ihfB**  message levels declined as cells entered the stationary growth phase.  Ref: 9714829 Gene, 1998
- The ihf genes  **ihfA**  and  **ihfB**  were cloned from N. gonorrhoeae through functional complementation of defined Escherichia coli ihf mutants for plating of phage lambda.  Ref: 9714829 Gene, 1998
- Protein IHF bound to two sites  **ihfA**  and  **ihfB**  in R388 oriT, as shown by gel retardation and DNase I footprinting analysis.  Ref: 10209739 Mol Microbiol, 1999
